# Cross-Regulations among NRFs and KEAP1 and Effects of their Silencing on Arsenic-Induced Antioxidant Response and Cytotoxicity in Human Keratinocytes

**DOI:** 10.1289/ehp.1104580

**Published:** 2012-01-03

**Authors:** Rui Zhao, Yongyong Hou, Qiang Zhang, Courtney G. Woods, Peng Xue, Jingqi Fu, Kathy Yarborough, Dawei Guan, Melvin E. Andersen, Jingbo Pi

**Affiliations:** 1 School of Forensic Medicine, China Medical University, Shenyang, China; 2 Institute for Chemical Safety Sciences, Hamner Institutes for Health Sciences, Research Triangle Park, North Carolina, USA

**Keywords:** antioxidant response, arsenic, cytotoxicity, KEAP1, keratinocyte, NRF1, NRF2

## Abstract

Background: Nuclear factor E2-related factors (NRFs), including NRF2 and NRF1, play critical roles in mediating the cellular adaptive response to oxidative stress. Human exposure to inorganic arsenic, a potent oxidative stressor, causes various dermal disorders, including hyperkeratosis and skin cancer.

Objective: We investigated the cross-regulations among NRF2, NRF1, and KEAP1, a cullin-3–adapter protein that allows NRF2 to be ubiquinated and degraded by the proteasome complex, in arsenic-induced antioxidant responses.

Results: In human keratinocyte HaCaT cells, selective knockdown (KD) of *NRF2* by lentiviral short hairpin RNAs (shRNAs) significantly reduced the expression of many antioxidant enzymes and sensitized the cells to acute cytotoxicity of inorganic arsenite (iAs^3+^). In contrast, silencing *KEAP1* led to a dramatic resistance to iAs^3+^-induced apoptosis. Pretreatment of HaCaT cells with NRF2 activators, such as *tert*-butylhydroquinone, protects the cells against acute iAs^3+^ toxicity in an NRF2-dependent fashion. Consistent with the negative regulatory role of KEAP1 in NRF2 activation, *KEAP1*-KD cells exhibited enhanced transcriptional activity of NRF2 under nonstressed conditions. However, deficiency in KEAP1 did not facilitate induction of NRF2-target genes by iAs^3+^. In addition, *NRF2* silencing reduced the expression of KEAP1 at transcription and protein levels but increased the protein expression of NRF1 under the iAs^3+^-exposed condition. In contrast, silencing *KEAP1* augmented protein accumulation of NRF2 under basal and iAs^3+^-exposed conditions, whereas the iAs^3+^-induced protein accumulation of NRF1 was attenuated in *KEAP1*-KD cells.

Conclusions: Our studies suggest that NRF2, KEAP1, and NRF1 are coordinately involved in the regulation of the cellular adaptive response to iAs^3+^-induced oxidative stress.

Arsenic (As) is a natural element that is ubiquitous in the environment in both organic and inorganic forms. Human exposure to inorganic arsenic (iAs), the more toxic form, occurs in environmental and occupational settings, as well as through medicinal arsenical use (Abernathy et al. 1999; Aposhian and Aposhian 2006). The skin is one of the most sensitive organs to chronic iAs toxicity. Arsenic-induced nonmalignant skin lesions, including hyperkeratosis and dyspigmentation, are some of the most common and earliest signs of chronic iAs exposure (Pi et al. 2000; Yoshida et al. 2004). Skin cancers associated with human iAs exposure include squamous cell carcinoma, basal cell carcinoma, intraepidermal carcinoma, and Bowen’s disease (carcinoma in situ) (Alain et al. 1993; International Agency for Research on Cancer 2004; Schwartz 1996; Wong et al. 1998). Although As is a well-established human skin toxicant, the underlying molecular mechanism(s) is still unclear. Accumulating evidence reveals that oxidative stress occurs in response to inorganic arsenite (iAs^3+^) exposure (Pi et al. 2002, 2003b; Zhao et al. 2011), which may account for the dermal toxicity induced by iAs^3+^, including hyperkeratosis and carcinogenesis.

Nuclear factor erythroid-derived factor 2–related factor 2 (NRF2), which belongs to the cap ’n’ collar (CNC) subfamily of basic-region leucine zipper (bZIP) transcription factors, is a central regulator in the cellular adaptive antioxidant response to oxidative stress (Villeneuve et al. 2010; Zhang 2010). The CNC subfamily also includes NRF1 (NFE2L1/LCRF1/TCF11), NRF3 (NFE2L3), and the nuclear factor-erythroid 2 p45 subunit, as well as more distantly related factors such as BTB and CNC homology 1 (BACH1) and BACH2 protein. Under nonstressed conditions, NRF1 is targeted to the endoplasmic reticulum (Biswas and Chan 2010), whereas NRF2 is primarily in the cytoplasm. When cells are under oxidative stress, NRF2 and/or NRF1 dimerize with small Maf or other bZIP proteins in the nucleoplasm, and the heterodimer binds to the *cis*-acting element(s) called antioxidant response elements (AREs) in the promoters of target genes, leading to their transcriptional activation (Biswas and Chan 2010; Maher and Yamamoto 2010). Kelch-like ECH-associated protein 1 (KEAP1), a cytoplasmic protein, serves as a substrate-adaptor molecule for cullin-3–based ubiquitin E3 ligase (Kobayashi et al. 2004; Zhang 2010). Under nonstressed conditions, KEAP1 binds to NRF2 and represses the activity of NRF2 by promoting its ubiquitination and proteasome-dependent degradation (Kobayashi et al. 2004; Villeneuve et al. 2010). When cells are exposed to electrophiles or oxidants, modification of the sulfhydryl groups in KEAP1 occurs, liberating NRF2 from KEAP1-mediated degradation, and NRF2 accumulates in the nucleus (Dinkova-Kostova et al. 2002; Motohashi and Yamamoto 2004). Fibroblasts derived from *Nrf1*-mutant mouse embryos and human keratinocytes with *NRF1* knockdown (KD) showed reduced glutathione (GSH) levels and higher sensitivity to the toxic effects of oxidants, including iAs^3+^ (Kwong et al. 1999; Zhao et al. 2011), suggesting a critical role for NRF1 in cellular oxidative defense. Although previous studies have suggested that NRF1 interacts with KEAP1 (Biswas and Chan 2010; Zhao et al. 2011), the biological significance of this interaction is still poorly characterized. In particular, the cross-regulations among NRF2, KEAP1, and NRF1 under oxidative stress conditions have not been fully investigated.

Previous studies (Aono et al. 2003), including our own (Pi et al. 2003b), suggested that NRF2 is a key player in the cellular adaptive response to iAs^3+^-induced oxidative stress, whereas constitutive NRF2 activation may be involved in arsenic carcinogenesis (Pi et al. 2008a). Our recent studies also demonstrated that long isoforms of NRF1 contribute to iAs^3+^-induced antioxidant response in human keratinocytes and protect the cells from acute arsenic cytotoxicity (Zhao et al. 2011). In the present study, the roles of NRF2 and KEAP1 in iAs^3+^-induced cytotoxicity and apoptosis, as well as the cross-regulations among NRF2, KEAP1, and NRF1 in response to acute iAs^3+^ exposure, were studied by using human keratinocyte HaCaT cells with selective knockdown of *NRF2* and *KEAP1* by lentiviral delivery of short hairpin RNAs (shRNAs). We demonstrated that cross-talk among NRF2, KEAP1, and NRF1 is an important part of iAs^3+^-induced antioxidant response and contributes to the coordinate regulation of antioxidant and detoxification enzyme expression, thus protecting cells from arsenic-induced apoptosis and cytotoxicity. Together with our previous findings (Zhao et al. 2011), we propose a comprehensive regulatory mechanism for iAs^3+^-induced adaptive antioxidant response in target cells of arsenic exposure, which may help advance our understanding of the mechanisms by which arsenic causes skin disorders.

## Materials and Methods

*Reagents and cell culture.* Sodium arsenite and *tert*-butylhydroquinone (tBHQ) were obtained from Sigma (St. Louis, MO, USA). The HaCaT cell is a spontaneously immortalized human epithelial cell line developed by Boukamp et al. (1988). The cells were cultured in Dulbecco’s modified Eagle’s medium supplemented with 10% fetal bovine serum (FBS), 100 U penicillin/mL, and 100 μg streptomycin/mL, as described previously (Pi et al. 2003b). Cultures were maintained at 37°C in a humidified 5% CO_2_ atmosphere. Culture media, FBS, and supplements were purchased from Invitrogen (Carlsbad, CA, USA).

*Lentiviral-based shRNA transduction.* MISSION shRNA lentiviral particles were obtained from Sigma. Transduction of HaCaT cells with lentiviral-based shRNAs targeting NRF2 (SHVRS-NM_006164), KEAP1 (SHVRS-NM_012289), or scrambled nontarget negative control (SHC002V) was performed as described previously (Zhao et al. 2011). Cells were maintained in medium containing 1.0 μg/mL puromycin.

*Quantitative real-time reverse-transcriptase polymerase chain (RT-PCR) reaction analysis.* Total RNA was isolated with TRIzol (Invitrogen) and then subjected to cleanup by using the RNase-Free DNase Set and the RNeasy Mini kit (Qiagen, Valencia, CA, USA). Quantitative real-time RT-PCR was performed as described previously (Zhao et al. 2011). Primers [sequences are shown in Supplemental Material, Table 1 (http://dx.doi.org/10.1289/ehp.1104580)] were designed by using Primer Express 4 (Applied Biosystems, Carlsbad, CA, USA) and synthesized by MWG Biotech Inc. (High Point, NC, USA). Real-time fluorescence detection was carried out by using an Applied Biosystems 7900HT Fast Real-Time PCR System (Applied Biosystems).

*Western blot analysis.* Protein isolation from whole-cell lysates and Western blotting were performed as detailed previously (Pi et al. 2003b; Zhao et al. 2011). Antibodies for NRF2 (sc-13032; 1:500), KEAP1 (sc-15246; 1:500), NRF1 (sc-13031; 1:500), and heme oxygenase 1 (HMOX-1; sc-136902; 1:500) were purchased from Santa Cruz Biotechnology Inc. (Santa Cruz, CA, USA). Antibody for NAD(P)H:quinone oxidoreductase 1 (NQO1; 39-3700; 1:1,000) was purchased from Invitrogen. Antibodies for β-ACTIN (A1978; 1:2,000) and glutamate-cysteine ligase, catalytic subunit (GCLC; RB-1697; 1:800) were from Sigma and Lab Vision (Fremont, CA, USA), respectively.

*Measurement of intracellular GSH.* Cells were sonicated in cold phosphate-buffered saline immediately after collection, followed by centrifugation at 12,000 × *g* for 5 min. The resulting supernatants were used for measurement of total GSH by using the BIOXYTECH GSH/GSSG-412 kit (OxisResearch, Portland, OR, USA) (Fu et al. 2010).

*Acute cytotoxicity assay.* A minimum of five replicates of 10,000 cells per well were plated in 96-well plates and allowed to adhere to the plate for 24 hr, at which time the media were removed and replaced with fresh media containing arsenic compounds. Cells were then incubated for an additional 24 hr, and cell viability was determined by using the Non-Radioactive Cell-Proliferation Assay Kit (Promega, Madison, WI, USA) as detailed previously (Zhao et al. 2011). Measurements were expressed as percentage change from untreated control (vehicle) of appropriate cells. The concentrations that were lethal to 50% of cells (LC_50_) were determined from analysis of the log-linear phase of the curves.

*Determination of apoptosis by flow cytometry.* Cells were seeded in six-well plates and cultured to approximately 80% confluence. Twenty hours after iAs^3+^ exposure, the cells, including floating and attached cells, were harvested for apoptosis analysis. Detection of phosphatidylserine on the outer leaflet of apoptotic cells was performed using TACS Annexin V-FITC (fluorescein isothiocynate) Apoptosis Detection Kit (Trevigen, Gaithersburg, MD, USA) as detailed previously (Pi et al. 2005). For each sample, 10,000 cells were examined by flow cytometry (FACS Vantage; Becton Dickinson, San Jose, CA, USA). Percentage of apoptotic cells (annexin V positive) was determined by statistical analysis of the various dot plots with the BD FACS Diva, version 6.1.2 (BD Biosciences, Bedford, MA, USA).

*Statistical analyses.* All statistical analyses were performed using Graphpad Prism 4 (GraphPad Software, San Diego, CA, USA), with *p* < 0.05 taken as significant. Data are expressed as mean ± SE. Statistical analyses to evaluate the effect of shRNAs on the gene expression of *NRF2* and *KEAP1* and intracellular GSH levels were carried out by using one-way analysis of variance (ANOVA) with Tukey’s or Dunnett’s multiple comparison test. Statistical analyses to evaluate the time- and concentration-dependent effect of iAs^3+^ exposure on gene expression and cell viability were performed by using two-way ANOVA with Bonferroni post hoc testing.

## Results

**Figure 1:**
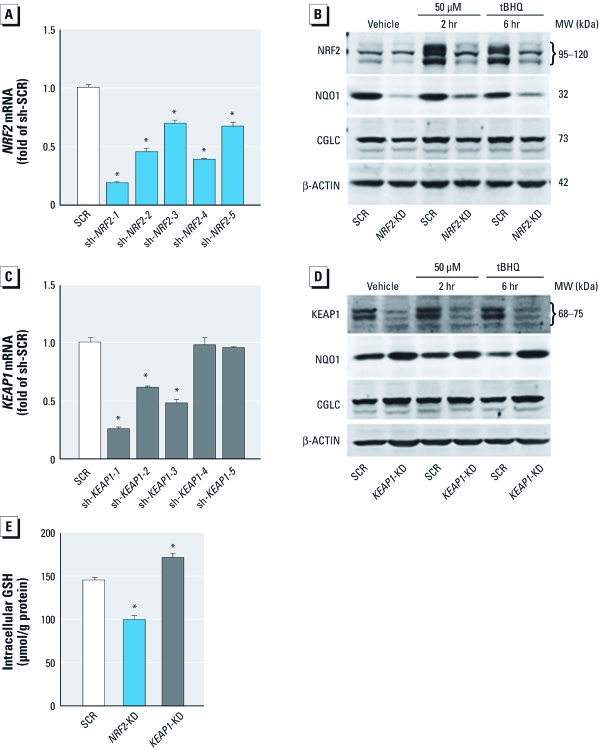
Stable knockdown of *NRF2* and *KEAP1* by lentiviral shRNAs in HaCaT cells. (*A* and *C*) Silencing effect of *NRF2* (*A*) and *KEAP1* (*C*) in HaCaT cells transduced with shRNA lentivirus targeted against human *NRF2*, *KEAP1*, or scrambled (SCR) nontarget negative control (sh-SCR) (*n* = 3). sh-NRF2-1 through sh‑NRF2-5 indicate shRNAs targeting *NRF2*; sh-KEAP1-1 through sh-KEAP1-5, shRNAs targeting *KEAP1*. (*B* and *D*) Protein expression of NRF2 (*B*), KEAP1 (*D*), and NRF2 target genes *NQO1* and *GCLC* in *NRF2*-KD and *KEAP1*-KD cells. Cells were treated with 50 μM tBHQ for the indicated time, and whole-cell lysates were separated on 4–12% Tris-glycine gels. β-ACTIN was used as loading control. (*E*) Intracellular GSH content in *NRF2*-KD and *KEAP1*-KD cells (*n* = 6). *p < 0.05 versus SCR (*A, C, E*).

*Stable knockdown of* NRF2 *and* KEAP1 *in HaCaT cells.* To study the role of NRF2-mediated antioxidant response in iAs^3+^-induced cytotoxicity, we performed lentiviral shRNA-mediated knockdown of *NRF2* and *KEAP1* in HaCaT cells. As shown in Figure 1, five shRNAs were used to target each of the two genes. One construct (sh-*NRF2-1*) markedly silenced *NRF2* expression compared with the scrambled nontarget negative control (SCR), whereas the other four *NRF2* constructs had moderate silencing effects (Figure 1A). The effectiveness of knockdown by sh-*NRF2-1* (*NRF2*-KD) was further confirmed by notably diminished protein accumulation of NRF2 when induced by tBHQ, a potent NRF2 activator (Pi et al. 2007). Our previous data indicated that two phosphorylated forms of human NRF2 near 95–120 kDa accumulate in the nucleus after chemically induced oxidative stress, including acute iAs^3+^ exposure (Pi et al. 2007). In the present study, both of the NRF2 bands were diminished by lentiviral shRNA-mediated knockdown, confirming that the silencing is effective and providing additional support for our previous findings (Pi et al. 2007). In addition, the protein expression of ARE-dependent genes *NQO1* and *GCLC* was attenuated, indicating that the transcriptional activity of NRF2 was suppressed in the *NRF2*-KD cells (Figure 1B).

Similarly, HaCaT cells transduced with construct sh-*KEAP1-1* (*KEAP1*-KD) showed the most efficient knockdown of *KEAP1* expression (Figure 1C,D). Supporting the silencing effect of *KEAP1*, enhanced expression of NQO1 and GCLC was observed in *KEAP1*-KD cells with or without tBHQ treatment (Figure 1D). Based on the Ensembl database, the human *KEAP1* has two splice variant transcripts coding for the same protein of 624 amino acids with a predicted molecular weight (MW) of 69.7 kDa. However, our immunoblots (Figure 1D) using an antibody developed against a peptide mapped near the N-terminus of human KEAP1 revealed two bands with apparent MW between 68 and 75 kDa. *KEAP1*-KD dramatically diminished both bands, suggesting that both represent endogenous human KEAP1. Clearly, additional research is required to characterize the two isoforms of KEAP1.

The key enzymes that regulate *de novo* GSH synthesis, including GCLC, glutamate-cysteine ligase, modifier subunit (GCLM), and glutathione synthetase, are well-documented ARE-dependent genes. Thus, intracellular GSH is an important indicator for the transcriptional activity of NRF2. As shown in Figure 1E, the intracellular GSH level in *NRF2*-KD cells was significantly decreased, whereas *KEAP1*-KD cells exhibited a modest but statistically significant increase in the GSH level compared with that of SCR cells, confirming the silencing effect of *NRF2* and *KEAP1*.

**Figure 2:**
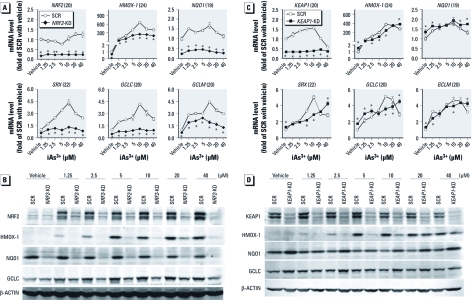
Effect of stable knockdown of *NRF2* or *KEAP1* in HaCaT cells on the induction of ARE-dependent genes in response to acute iAs^3+^ exposure. Cells were exposed to iAs^3+^ or vehicle (medium) for 6 hr. (*A*, *B*) Concentration response of iAs^3+^-induced mRNA (*A*) and protein (*B*) expression of NRF2 and ARE-dependent antioxidant genes in *NRF2*-KD and SCR cells. (*C*, *D*) Concentration response of iAs^3+^-induced mRNA (*C*) and protein (*D*) expression of KEAP1 and ARE-dependent antioxidant genes in *KEAP1*-KD and SCR cells. Integers in parentheses after gene names in *A* and *C* are cross-threshold PCR cycle numbers in SCR cells treated with vehicle. *p < 0.05 versus SCR with the same treatment.

*Stable knockdown of* NRF2 *results in attenuated antioxidant induction in response to iAs^3+^ exposure.* To define the role of NRF2-mediated antioxidant response in cellular defense against iAs^3+^ toxicity, *NRF2*-KD and SCR cells were acutely exposed to iAs^3+^, and the expression of NRF2 and the ARE-dependent genes *HMOX-1*, *NQO1*, *GCLC*, *GCLM*, and sulfiredoxin (*SRX*) was determined [Figure 2A,B; see also Supplemental Material, Figure 1A,B (http://dx.doi.org/10.1289/ehp.1104580)]. In SCR cells, iAs^3+^ concentration- and time-dependently increased NRF2 and ARE-dependent gene expression at both mRNA (Figure 2A; see also Supplemental Material, Figure 1A) and protein (Figure 2B; see also Supplemental Material, Figure 1B) levels, confirming our previous findings that iAs^3+^ is a potent NRF2 activator (Pi et al. 2003b, 2007). In contrast, *NRF2*-KD cells exhibited dramatically attenuated expression of NRF2 and ARE-dependent genes under both basal and iAs^3+^-exposed conditions (Figure 2A,B; see also Supplemental Material, Figure 1A,B).

*Stable knockdown of* KEAP1 *results in enhanced basal expression of ARE-dependent genes but weakens iAs^3+^-induced antioxidant response.* Consistent with the inhibitory role of KEAP1 in regulating NRF2-ARE activity, *KEAP1*-KD cells exhibited enhanced expression of the ARE-dependent genes *HMOX-1*, *NQO1*, *SRX1*, *GCLC*, and *GCLM* under basal conditions [Figure 2C,D; see also Supplemental Material, Figure 1C,D (http://dx.doi.org/10.1289/ehp.1104580)]. In apparent contrast to the enhanced basal expression, deficiency in KEAP1 diminished iAs^3+^-induced expression of HMOX-1 at mRNA and protein levels (Figure 2C,D; see also Supplemental Material, Figure 1C,D). In addition, no constant increase in iAs^3+^-induced expression of other ARE-dependent genes was observed in *KEAP1*-KD cells. These findings suggest that constitutive activation of NRF2 may weaken the induction of ARE-dependent genes, in particular *HMOX-1*, in response to acute iAs^3+^ exposure. Note that acute exposure (6 hr) to high concentrations of iAs^3+^ (20–40 μM) suppressed KEAP1 expression in SCR cells (Figure 2C,D), which might contribute to high-concentration iAs^3+^-induced NRF2 activation.

**Figure 3:**
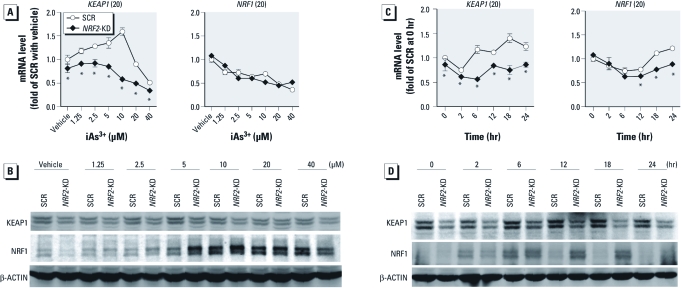
Stable knockdown of *NRF2* in HaCaT cells reduces expression of KEAP1 but augments iAs^3+^-induced protein accumulation of long isoforms of NRF1. (*A*, *B*) Concentration response of mRNA (*A*) and protein (*B*) expression of KEAP1 and NRF1 in *NRF2*-KD and SCR cells exposed to various concentrations of iAs^3+^ or vehicle (medium) for 6 hr. (*C,* *D*) Time course of mRNA (*C*) and protein (*D*) expression of KEAP1 and NRF1 in *NRF2*-KD and SCR cells in response to 10 μM iAs^3+^ or vehicle. Integers in parentheses after gene names in *A* and *C* are cross-threshold PCR cycle numbers in SCR cells treated with vehicle. *p < 0.05 versus SCR with the same treatment.

**Figure 4:**
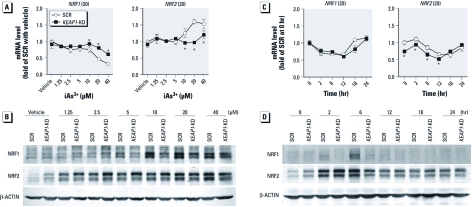
Effect of stable knockdown of *KEAP1* in HaCaT cells on the expression of NRF1 and NRF2 in response to iAs^3+^ exposure. (*A*, *B*) Concentration response of mRNA (*A*) and protein (*B*) expression of NRF1 and NRF2 in *KEAP1*-KD and SCR cells exposed to iAs^3+^ or vehicle (medium) for 6 hr. (*C*, *D*) Time course of mRNA (*C*) and protein (*D*) expression of NRF1 and NRF2 in *NRF2*-KD and SCR cells in response to 10 μM iAs^3+^ or vehicle. Integers in parentheses after gene names in *A* and *C* are cross-threshold PCR cycle numbers in SCR cells treated with vehicle. *p < 0.05 versus SCR with the same treatment.

*Cross-regulations among* NRF2, KEAP1, *and* NRF1 *in iAs^3+^-induced antioxidant response in HaCaT cells.* Although the manner in which NRF2 is regulated by KEAP1 has been well documented, the effect of NRF2 deficiency on expression of KEAP1 is not clear, in particular under oxidative stress conditions. As shown in Figure 3, lack of NRF2 resulted in attenuated gene and protein expression of KEAP1 under basal and iAs^3+^-exposed conditions, suggesting that the expression of KEAP1 is dependent on NRF2. Although deficiency of NRF2 did not increase the mRNA expression of *NRF1* (Figure 3A,C), *NRF2*-KD cells exhibited increased protein accumulation of NRF1 under high concentrations of iAs^3+^ (2.5–20 μM for 6 hr and 10 μM for 6–24 hr), suggesting that NRF1 may compensate for the deficiency of NRF2 under iAs^3+^-exposed conditions to protect the cells from oxidative damage. In addition, an induction in NRF1 protein levels was observed in SCR cells but was short-lived compared with the sustained response observed in *NRF2*-KD cells (Figure 3D). In contrast, knockdown of *KEAP1* augmented the protein accumulation of NRF2 under basal and iAs^3+^-exposed conditions (Figure 4). Conversely, *KEAP1*-KD cells exhibited reduced protein level, but not mRNA expression, of NRF1 induced by high concentrations of iAs^3+^ (> 5 μM; Figure 4). The weakened protein accumulation of NRF1 in response to high concentrations of iAs^3+^ in *KEAP1*-KD cells could result from enhanced NRF2-mediated antioxidant response, leading to augmented detoxification capacity and/or lowered intracellular iAs^3+^ level. The difference between mRNA and protein expression of NRF1 suggests that protein stabilization is a major mechanism for NRF1 activation, which is consistent with a recent report (Steffen et al. 2010).

**Figure 5:**
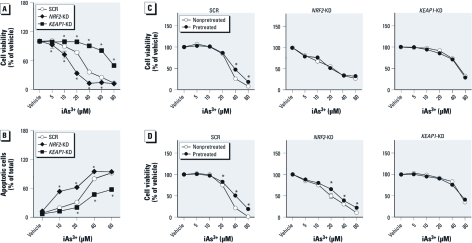
iAs^3+^-induced cytotoxicity and apoptosis in *NRF2*-KD and *KEAP1*-KD HaCaT cells. (*A*) *NRF2*-KD cells are sensitive, whereas *KEAP1*-KD cells are resistant, to iAs^3+^-induced cytotoxicity. (*B*) Quantification of iAs^3+^-induced apoptotic (annexin V-positive) cells by flow cytometry. Cells were exposed to various concentrations of iAs^3+^ for 20 hr (*n* = 3). (*C* and *D*) Effects of pretreatment with noncytotoxic, low-level NRF2 activators on cytotoxicity induced by subsequent exposure to high concentrations of iAs^3+^ in SCR, *NRF2*-KD, or *KEAP1*-KD HaCaT cells. HaCaT cells were pretreated with 25 μM tBHQ (*C*) or 2.5 μM iAs^3+^ (*D*) for 6 hr, followed by iAs^3+^ exposure for 24 hr. (*n* = 6). *p < 0.05 versus SCR treated with the same concentration of iAs^3+^.

*Distinct effect of knockdown of* NRF2 *or* KEAP1 *on iAs^3+^-induced cytotoxicity and apoptosis in HaCaT cells.* To investigate the roles of NRF2 in iAs^3+^-induced cytotoxicity, acute (24 hr) effects of iAs^3+^ exposure on cell metabolic integrity were measured in *NRF2*-KD and *KEAP1*-KD HaCaT cells. As shown in Figure 5A, deficiency of *NRF2* significantly enhanced the cells’ sensitivity to iAs^3+^ toxicity. The LC_50_ value was 16.3 ± 2.5 μM in *NRF2*-KD cells versus 36.0 ± 2.1 μM in SCR cells (*p* < 0.05). In contrast, knockdown of *KEAP1* led to a dramatic resistance to iAs^3+^ toxicity (Figure 5A). The LC_50_ value in the *KEAP1*-KD cells was 80.1 ± 3.1 μM, which was significantly higher than that of SCR. We then measured iAs^3+^-induced apoptosis and necrosis using flow cytometry with annexin V-FITC and propidium iodide double staining. Consistent with the results of cytotoxicity, knockdown of *NRF2* in HaCaT cells dramatically enhanced the sensitivity to iAs^3+^-induced apoptosis, whereas deficiency of *KEAP1* led to a significant apoptotic resistance (Figure 5B).

To provide further support of our hypothesis that NRF2 activation contributes to iAs^3+^- induced apoptotic resistance, the effect of pretreatment of HaCaT cells with NRF2 activator tBHQ on iAs^3+^-induced cytotoxicity was examined. As shown in Figure 5C, pretreatment of SCR cells with noncytotoxic level of tBHQ (25 μM) for 6 hr modestly but statistically significantly protected the cells from subsequent cytotoxicity induced by high concentrations of iAs^3+^. However, pretreatment of *NRF2*-KD cells with tBHQ showed no protective effect against iAs^3+^ toxicity, suggesting that the protective effect of tBHQ pretreatment is a result of NRF2 activation. In addition, no further protective effect was observed by tBHQ pretreatment in *KEAP1*-KD cells, possibly because NRF2 is already maximally activated in these cells and cannot be increased further by tBHQ. In contrast, the protective effect of pretreatment with noncytotoxic dose of iAs^3+^ (2.5 μM) revealed a somewhat different pattern. Although iAs^3+^ pretreatment can protect against cytotoxicity subsequently induced by higher doses of iAs^3+^ in SCR cells, the protection also exists in *NRF2*-KD cells, suggesting that low-dose iAs^3+^ might trigger, in addition to NRF2, other adaptive mechanisms, such as activation of NRF1 (Figure 5D). Like tBHQ pretreatment, iAs^3+^ pretreatment did not provide further protection of *KEAP1*-KD cells from high-dose iAs^3+^ toxicity. The finding that NRF2 activators exhibited less significant effect than *KEAP1* silencing on cell survival after arsenic treatment suggests that knockdown of *KEAP1* resulted in a higher level of antioxidant response than did tBHQ or arsenic pretreatment and/or that *KEAP1* silencing also activated an NRF2-independent mechanism to contribute to the protection.

## Discussion

Supporting the importance of NRF2 in cellular defense is the finding that Nrf2-knockout mice show deficiency in the coordinated antioxidant and phase II gene induction program and have a higher susceptibility to both oxidative damage and chemical carcinogenesis (Chan et al. 2001; Ramos-Gomez et al. 2001). In addition, lack of NRF2 in various mammalian cells has been reported to be associated with heightened sensitivity to various oxidative insults, including iAs-induced and ultraviolet A–induced apoptosis (Calkins et al. 2005; Hirota et al. 2005; Lee et al. 2003). In the present study, we provide evidence that NRF2 regulates many ARE-dependent genes and contributes to resistance to iAs^3+^-induced cytotoxicity and apoptosis in human keratinocytes. In apparent contrast to extensive data showing that lowered NRF2 activity predisposes cells to chemical carcinogenesis, emerging evidence reveals the dichotomy that constitutive activation of NRF2 may contribute to malignant phenotypes (Padmanabhan et al. 2006). Indeed, increased expression and activity of NRF2, resulting from mutations in KEAP1 and/or NRF2, have been observed in various tumor cells, including skin, breast, prostate, lung, head/neck, and endometrium (Kim et al. 2010; Ohta et al. 2008; Padmanabhan et al. 2006; Shibata et al. 2008; Singh et al. 2006; Stacy et al. 2006). In humans, NRF2 activation due to *NRF2* or *KEAP1* gene mutation plays an important role in the development of skin squamous cell carcinoma (Kim et al. 2010), which is the most common type of skin cancer caused by chronic iAs exposure. Most recently, DeNicola et al. (2011) reported that oncogene-induced *NRF2* transcription promotes reactive oxygen species (ROS) detoxification and tumorigenesis. These seemingly contradictory findings suggest that NRF2 may play paradoxical roles in different stages of tumorigenesis. On the one hand, NRF2-dependent antioxidant and detoxification enzymes may promote the detoxification and elimination of ROS and other carcinogens to alleviate their carcinogenic effect. On the other hand, NRF2 activation may provide cell survival advantage by contributing to acquired apoptotic resistance, which is an important event in the process of arsenic-induced malignant transformation in many cell types, including human keratinocytes (Pi et al. 2005, 2008b; Tokar et al. 2010). Therefore, the timing and cellular target of intervention targeting NRF2 are critically important for chemoprevention of cancers.

Hyperkeratosis is one the most common human skin disorders caused by chronic iAs exposure (Pi et al. 2000; Wong et al. 1998; Yoshida et al. 2004). The underlying mechanism is, however, poorly understood. iAs^3+^ has been identified as a potent activator of NRF2-mediated antioxidant response (Pi et al. 2003a), whereas NRF2 and its target genes have been reported as important regulators in skin-wound healing (Braun et al. 2002) and arsenic-induced skin injury (Pi et al. 2003b, 2008a). In the present study, we confirmed the stimulatory effect of iAs^3+^ on NRF2 activation in human keratinocytes and demonstrated that high concentrations of iAs^3+^ inhibit the expression of KEAP1, which may contribute to NRF2 activation by iAs^3+^. It has been reported that disruption of *Keap1* in mice leads to hyperkeratosis in esophagus, forestomach, and skin, most likely because of constitutive activation of NRF2 and aberrant expression of some ARE-dependent cytokeratins (Wakabayashi et al. 2003). Therefore, iAs^3+^-induced persistent activation of NRF2 may be associated with the pathogenesis of skin hyperkeratosis, although additional studies are needed to confirm the linkage.

The distinctive roles of NRF2 and NRF1 in arsenic-induced keratinocyte toxicity have been demonstrated in our previous studies (Pi et al. 2003b, 2005, 2007, 2008a; Zhao et al. 2011). However, the cross-regulations among NRF2, NRF1, and KEAP1 in iAs^3+^-induced antioxidant response have not been investigated. In the present study, we observed higher levels of NRF1 protein accumulation in *NRF2*-KD cells than of SCR in response to high concentrations of iAs^3+^ exposure, suggesting that NRF1 may compensate for deficiency of NRF2 in iAs^3+^-induced antioxidant response. This is consistent with the finding by Braun et al. (2002) demonstrating that mice lacking NRF2 had no significant wound healing phenotype partially because of an up-regulation of NRF1. In contrast, silencing of *NRF1* in HaCaT cells does not interfere with iAs^3+^-induced NRF2 protein accumulation, revealing that NRF2 cannot compensate for deficiency of NRF1 in iAs^3+^-induced antioxidant response (Zhao et al. 2011). In addition, reduced KEAP1 expression was observed in *NRF1*-KD HaCaT cells, suggesting a potential regulation of KEAP1 by NRF1 (Zhao et al. 2011). In the present study, we found that lack of NRF2 also led to reduced expression of KEAP1. Taken together, the reduction in expression of KEAP1 in both *NRF1*- and *NRF2*-KD cells highly suggests that *KEAP1* is an ARE-dependent gene. It is unclear whether such regulation, if indeed present, has any functional significance.

In contrast to increased expression of NRF2 and its target genes under basal conditions, a weakened induction of HMOX-1 by iAs^3+^ was observed in *KEAP1*-KD cells. This finding is consistent with our previous study showing that persistent activation of NRF2 by chronic iAs^3+^ exposure paradoxically represses NRF2-mediated antioxidant induction in response to additional acute oxidative challenges (Pi et al. 2008a). In addition, *KEAP1*-KD cells exhibited reduced protein levels of NRF1 induced by high concentrations of iAs^3+^, suggesting a potential association between NRF1 and KEAP1. Our previous study indicated that exogenous GSH markedly suppresses hypochlorous acid-induced NRF2 activation in mouse macrophages (Pi et al. 2008a), suggesting that the enhanced GSH levels in *KEAP1*-KD cells may be a critical factor for the weakened NRF2 activation. In addition, elevated levels of NRF2 and subsequent induction of phase II enzymes in *KEAP1*-KD cells might reduce the intracellular concentration of iAs^3+^ and thus result in decreased activation of NRF2 and NRF1.

In summary, we provide evidence that NRF2, KEAP1, and NRF1 interact with each other and coordinate regulation of cellular adaptive antioxidant responses to acute iAs^3+^ exposure. Given the potential importance of oxidative stress in arsenic-induced dermal toxicity and carcinogenicity, as well as the critical roles of NRF2 and NRF1 in defending against oxidative damage and the pathogenesis of skin cancer and hyperkeratosis, our findings provide an important insight into the mechanism for dermal toxicity induced by arsenic exposure.

## Supplemental Material

(352 KB) PDF
