# Supplementary material for: Cross-Regulations among NRFs and KEAP1 and Effects of their Silencing on Arsenic-Induced Antioxidant Response and Cytotoxicity in Human Keratinocytes
Source: Environ Health Perspect. 2012 Jan 3;120(4):583–9. doi: 10.1289/ehp.1104580 (PMC3339469; doi:10.1289/ehp.1104580)
Supplement: (352 KB) PDF [file ehp.1104580.s001.pdf]

Supplemental Material

Cross-Regulations among NRFs and KEAP1 and Effects of Their Silencing on Arsenic-Induced Antioxidant Response and Cytotoxicity in Human Keratinocytes

Rui Zhao, Yongyong Hou, Qiang Zhang, Courtney G. Woods, Peng Xue, Jingqi Fu, Kathy Yarborough, Dawei Guan, Melvin E. Andersen, Jingbo Pi

Contents of Supplemental Material

Table 1 -- Page 3

Figure 1 -- Page 4

Supplemental Material, Table 1. Primer sequences for Real-time RT-PCR

Gene name	GeneBank Accession	Position	Forward (5' - 3')	Reverse (5' - 3')
NRF2	NM-006164	398-488	AACCAGTGGATCTGCCAACTACTC	CTGCGCCAAAAGCTGCAT
KEAP1	NM_012289	2257-2342	CCTCTGGCCGGGTAATAGG	CCCCTCCCAGGTATCCAAGA
NRF1	NM_003204	4172-4259	GCCCTGTTTCACTTATAGGGTCTAGA	GGCAAAGAGAACATTTAGCAGCTT
HMOX1	NM_002133	1216-1294	GCCTGGAAGACACCCTAATGTG	GGCCGTGTCAACAAGGATACTT
NQO1	NM_000903	2154-2264	ACTGCCCTCTTGTGGTGCAT	GCTCGGTCCAATCCCTTCAT
SRX	NM_080725	326-400	GGAGGTGACTACTTCTACTCCTTTGG	GATGGTCTCTCGCTGCAGTTG
GCLC	NM_001498	1521-1625	GATGCTGTCTTGCAGGGAATG	AGCGAGCTCCGTGCTGTT
GCLM	NM_002061	830-910	ACAGGTAAAACCAAATAGTAACAAAGTTAA	TGTTTAGCAAATGCAGTCAAATCTG
β-ACTIN	X00351	1088-1063	GTCCACCTTCCAGCAGATGTG	GCATTTGCGGTGGACGAT

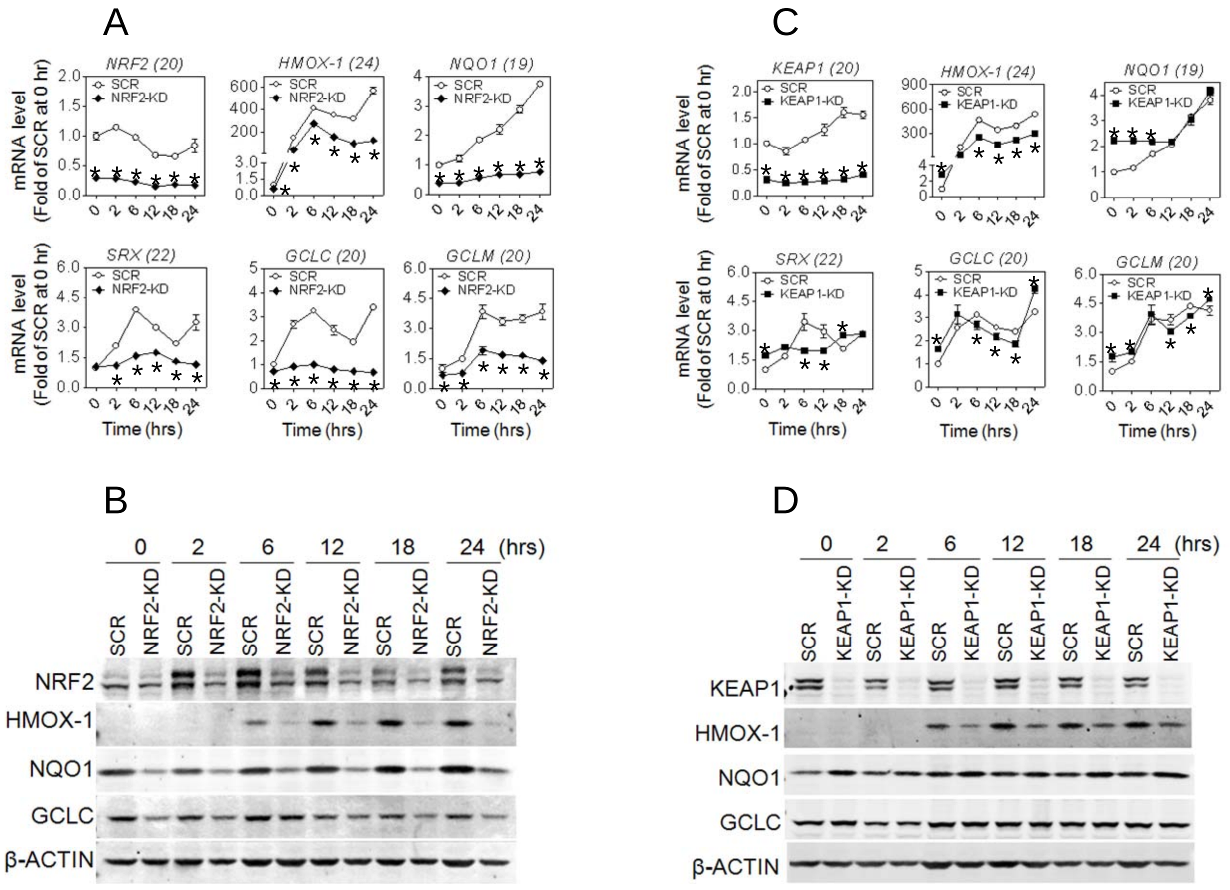

Supplemental Material, Figure 1. Time-course of iAs^{3+}-induced mRNA (*A* and *C*) and protein (*B* and *D*) expression of NRF2, KEAP1 and ARE-dependent antioxidant genes in *NRF2*-KD (*A* and *B*) and *KEAP1*-KD (*C* and *D*) cells. Cells were exposed to 10 μM iAs^{3+} or Vehicle for indicated times. *, $p < 0.05$ vs. SCR with the same treatment.
